# Spatiotemporal Expression of GRP78 in the Blood Vessels of Rats Treated With 3-Nitropropionic Acid Correlates With Blood–Brain Barrier Disruption

**DOI:** 10.3389/fncel.2018.00434

**Published:** 2018-11-20

**Authors:** Xuyan Jin, Tae-Ryong Riew, Hong Lim Kim, Soojin Kim, Mun-Yong Lee

**Affiliations:** ^1^Department of Anatomy, Catholic Neuroscience Institute, College of Medicine, The Catholic University of Korea, Seoul, South Korea; ^2^Department of Biomedicine and Health Sciences, College of Medicine, The Catholic University of Korea, Seoul, South Korea; ^3^Integrative Research Support Center, Laboratory of Electron Microscope, College of Medicine, The Catholic University of Korea, Seoul, South Korea

**Keywords:** 78-kDa glucose-regulated protein, striatum, endoplasmic reticulum, 3-nitropropionic acid, blood vessels, blood–brain barrier

## Abstract

Glucose-regulated protein (GRP78) or BiP, a 78-kDa chaperone protein located in the endoplasmic reticulum (ER), has recently been reported to be involved in the neuroglial response to ischemia-induced ER stress. The present study was designed to study the expression patterns of this protein and the cell types involved in the induction of GRP78 expression in rats treated with the mitochondrial toxin 3-nitropropionic acid (3-NP). GRP78 immunoreactivity was almost exclusively localized to striatal neurons in saline-treated controls, but GRP78 expression was induced in activated glial cells, including reactive astrocytes and activated microglia/macrophages, in the striata of rats treated with 3-NP. In the lesion core, increased GRP78 immunoreactivity was observed in the vasculature; this was evident in the lesion periphery of the core at 3 days after lesion induction, and was evenly distributed throughout the lesion core by 7 days after lesion induction. Vascular GRP78 expression was correlated, both temporally and spatially, with infiltration of activated microglia into the lesion core. In addition, this was coincident with the time and pattern of blood–brain barrier (BBB) leakage, detected by the extravasation of fluorescein isothiocyanate-albumin, an established BBB permeability marker. Vascular GRP78-positive cells in the lesion core were identified as endothelial cells, smooth muscle cells, and adventitial fibroblast-like cells, in which GRP78 protein was specifically localized to the cisternae of the rough ER and perinuclear cisternae, but not to other organelles such as mitochondria or nuclei. Thus, our data provide novel insights into the phenotypic and functional heterogeneity of GRP78-positive cells within the lesion core, suggesting the involvement of GRP78 in the activation/recruitment of activated microglia/macrophages and its potential role in BBB impairment in response to a 3-NP-mediated neurotoxic insult.

## Introduction

The 78-kDa glucose-regulated protein (GRP78), also known as the immunoglobulin heavy-chain binding protein or BiP, is a multifunctional regulator of endoplasmic reticulum (ER) homeostasis and stress response (Bole et al., 1989; Zhang and Zhang, 2010; Ouyang et al., 2011). ER stress triggers a cell stress response, termed the unfolded protein response, which activates the apoptosis pathway, when ER stress is excessive or prolonged (for review, see Zhu and Lee, 2015; Casas, 2017). GRP78, a major upregulated target protein in the unfolded protein response, regulates the folding and assembly of newly synthesized proteins, facilitates protein translocation across the ER membranes, and acts as a calcium binding protein (for review, see Zhu and Lee, 2015; Casas, 2017). In addition, GRP78 can translocate from the ER to the cytosol, nucleus, mitochondria, and plasma membrane, and it can also be secreted, suggesting its involvement in the regulation of cell viability and signaling (Sun et al., 2006; Gonzalez-Gronow et al., 2009; Liu et al., 2010; Misra and Pizzo, 2010a,b; Misra et al., 2011; Ni et al., 2011; Ouyang et al., 2011).

The neuronal functions of GRP78 have been investigated in various central nervous system (CNS) disease models, including ischemic insults (Wang et al., 1993; Aoki et al., 2001; Ito et al., 2001; Shibata et al., 2003; Tajiri et al., 2004; Oida et al., 2008; Osada et al., 2010), epileptic seizures (Wang et al., 1993; Chen et al., 2013), spinal cord injury (Penas et al., 2007, 2011; Ohri et al., 2012; Matsuyama et al., 2014), diabetic encephalopathy (Zhao et al., 2015), and experimental subarachnoid hemorrhage (Liu et al., 2016). GRP78 overexpression can have a neuroprotective effect by inhibiting the unfolded protein response, promoting autophagy, buffering calcium unbalance, and activating pro-survival signaling pathways (Ouyang et al., 2011; Zhang et al., 2015; Casas, 2017). Although most studies investigating GRP78 have focused on neuron-specific functions, there is increasing evidence of alterations in GRP78 expression in activated glial cells after CNS insults. GRP78 expression is induced in reactive astrocytes following status epilepticus and ischemia (Osada et al., 2010; Ko et al., 2011), and in microglia/macrophages after spinal cord injury (Fan et al., 2015). In addition, *in vitro* evidence indicates that GRP78 overexpression in astrocytes protects against ER stress (Ouyang et al., 2011; Suyama et al., 2011). Furthermore, our recent *in vivo* study showed prominent induction of GRP78 expression within activated glial cells after transient focal cerebral ischemia, predominantly in microglia/macrophages and reactive astrocytes (Jin et al., 2018a). Thus, the aforementioned data indicate a phenotypic and functional heterogeneity of GRP78-positive cells in the injured CNS, suggesting a multifunctional role, possibly in the neuroglial reaction to CNS insults, in addition to its known neuroprotective role. However, the detailed expression pattern of GRP78 and the cell types involved in the induction of GRP78 expression have been analyzed only in the ischemic brain. Thus, these findings need to be further substantiated in other models of CNS insults.

To address these issues, we examined the temporal changes and cellular localization of GRP78 expression in the lesioned striatum following injection of the natural mitochondrial toxin 3-nitropropionic acid (3-NP), which selectively damages medium-spiny striatal neurons and thus mimics many of the histological and neurochemical features characteristic of Huntington’s disease (Hamilton and Gould, 1987; Beal et al., 1993; Borlongan et al., 1997). This 3-NP model accurately mimics the dynamic spatiotemporal regulation of neuroglial activation in response to injuries, producing tissue lesions consisting of well-demarcated cores and perilesional areas with astroglial scar formation (Duran-Vilaregut et al., 2010; Mu et al., 2016; Riew et al., 2017).

## Materials and Methods

### Animal Preparation

All experimental procedures were conducted in accordance with the Laboratory Animal Welfare Act, the Guide for the Care and Use of Laboratory Animals, and Guidelines and Policies for Rodent Survival Surgery, and were approved by the Institutional Animal Care and Use Committee at the College of Medicine, The Catholic University of Korea (Approval Number: CUMC-2017-0321-04). All efforts were made to minimize animal suffering and to reduce the number of animals used.

Adult, male Sprague-Dawley rats (250–300 g, aged 9–11 weeks) were used in this study. Animals were housed in groups of three per cage in a controlled environment at a constant temperature (22 ± 5°C) and humidity (50 ± 10%) with food (gamma ray-sterilized diet) and water (autoclaved tap water) available *ad libitum*. They were maintained on a 12-h light/dark cycle. 3-NP (Sigma-Aldrich, St. Louis, MO, United States) was dissolved in buffered saline (pH 7.0), and administered intraperitoneally (i.p.) at a dose of 15 mg/kg once daily for 3 days. All 3-NP-injected rats were evaluated daily for the presence of behavioral deficits, and only rats exhibiting neurological deficit symptoms, including hind limb impairment or a kyphotic posture, recumbency, and impaired postural adjustments, were included in the experimental group (Hamilton and Gould, 1987). Animals were sacrificed 1, 3, 7, and 14 days after the final injection of 3-NP (*n* = 6/time point). The control group (*n* = 3) received intraperitoneal injections of the same volume of normal saline for three consecutive days and were sacrificed 3 days after the final injection. The animals were anesthetized with 10% chloral hydrate, sacrificed, and then perfused transcardially with 4% paraformaldehyde in 0.1 M phosphate buffer (PB; pH 7.4) The brain tissues were equilibrated with 30% sucrose in 0.1 M PB and frozen whole.

### Immunohistochemistry

For GRP78 immunohistochemistry, coronal cryostat sections (25-μm-thick) were incubated in blocking buffer solution (0.2% gelatin, 0.05% saponin, and 1% bovine serum albumin in phosphate-buffered saline) and then incubated overnight at 4°C with a rabbit polyclonal antibody to GRP78 (1:2000; Abcam, Cambridge, United Kingdom). Primary antibody binding was visualized using peroxidase-labeled goat anti-rabbit antibody (1:100; Jackson ImmunoResearch, West Grove, PA, United States) and 0.05% 3,3′, -diaminobenzidine tetrahydrochloride (DAB) with 0.01% H_2_O_2_ as a substrate. The specificity of GRP78 immunoreactivity was confirmed by the absence of immunohistochemical staining in sections from which the primary or secondary antibody had been omitted. Tissue sections were scanned and photographed using a slide scanner (SCN400, Leica Microsystems Ltd., Mannheim, Germany). Images were converted to TIFF format, and contrast levels adjusted using Adobe Photoshop v. 13.0 (Adobe Systems, San Jose, CA, United States).

For the evaluation of tissue injury, serial sections from sham controls and experimental rats at 3 days post-lesion were processed for Fluoro-Jade B (FJB) histochemistry and immunohistochemistry for GRP78. For FJB staining, sections were stained with 0.0004% FJB (Millipore, Temecula, CA, United States) in distilled water containing 0.01% acetic acid for 30 min according to the manufacturer’s protocol. After rinsing in distilled water, the sections were immersed in xylene and cover-slipped with DPX mounting medium (Sigma-Aldrich).

For triple-labeling, non-specific staining was blocked by preincubation of free-floating sections (25-μm-thick) in blocking buffer (3% normal goat serum, 1% bovine serum albumin, and 0.5% triton). Primary antibodies and dilutions were as follows: rabbit polyclonal antibody against GRP78 (1:2000; Abcam), mouse monoclonal antibody against rat endothelial cell antigen-1 (RECA1; 1:200; Bio-Rad, Hercules, CA, United States), mouse monoclonal antibodies against glial fibrillary acidic protein (GFAP; 1:700; Millipore), mouse monoclonal antibody against NeuN (1:500; Millipore), goat polyclonal antibody against ionized calcium-binding adaptor molecule 1 (Iba1; 1:500; Abcam), mouse monoclonal antibody against nestin (1:500; Millipore), goat polyclonal antibody against choline acetyltransferase (1:300; Millipore), mouse monoclonal antibodies to ED1 (1:50; Bio-Rad) or mouse monoclonal antibodies to CD45 (1:15; Bio-Rad). In addition, double labeling was performed using a mix of rabbit polyclonal antibody against GRP78 (1:2000; Abcam), and one of following antibodies: mouse monoclonal antibody to NG2 (1:500; Millipore) or to α-smooth muscle actin (α-SMA; 1:500; Sigma-Aldrich). This triple- or double-labeling was followed by a 2-h incubation with appropriate secondary antibodies, as follows: Cy3-conjugated donkey anti-goat antibody (1:2000; Jackson ImmunoResearch), Cy3-conjugated donkey anti-mouse antibody (1:2000; Jackson ImmunoResearch), Alexa Fluor 488-tagged donkey anti-rabbit antibody (1:300; Thermo Fisher, Waltham, MA, United States), Alexa Fluor 647-conjugated donkey anti-mouse antibody (1:300; Thermo Fisher), or Alexa Fluor 647-conjugated donkey anti-rabbit antibody (1:300; Thermo Fisher). Negative staining controls for the triple- or double-immunofluorescence were performed by omission of the primary or secondary antibodies. In addition, we compared the results of triple or double-labeling with those from single-labeling of all combinations of antibodies to ensure clear interpretation of results. Counterstaining of cell nuclei was carried out using DAPI (4′,6-diamidino-2-phenylindole; 1:2000; Roche, Mannheim, Germany) for 10 min.

In order to detect apoptotic cells simultaneously with GRP78 expression in neurons, we performed triple-labeling for terminal deoxynucleotidyl transferase dUTP nick end labeling (TUNEL) according to the manufacturer’s protocol (Roche Diagnostics Corporation, Indianapolis, IN, United States) and the following antibodies: rabbit polyclonal antibody against GRP78 (1:2000; Abcam) and mouse monoclonal antibody against NeuN (1:500; Millipore). This was followed by a 2-h incubation with Alexa Fluor 488-tagged goat anti-rabbit antibody (1:300; Thermo Fisher), Alexa Fluor 647-tagged goat anti-mouse antibody (1:300; Thermo Fisher), and Cy3-conjugated streptavidin (1:1200; Jackson ImmunoResearch) for the TUNEL method. Counterstaining of cell nuclei was carried out with DAPI for 10 min. Slides were viewed under a confocal microscope (LSM 700; Carl Zeiss Co., Ltd., Oberkochen, Germany) equipped with four lasers (Diode 405, Argon 488, HeNe 555, and HeNe 639) under constant viewing conditions. Images were converted to TIFF format, and contrast levels and sizes were adjusted using Adobe Photoshop v.13.0.

### Quantitative Analysis

To quantify time-dependent changes in GRP78 immunoreactivity associated with the vasculature after 3-NP injection, we analyzed the immunofluorescence intensities from the confocal data by using ZEN 2.1 Blue Edition software (Carl Zeiss Co., Oberkochen, Germany). Sections double-labeled for GRP78 and RECA1, a vascular endothelial cell marker, in sham-operated and experimental rats at days 1, 3, and 7 after reperfusion (*n* = 3 animals per time point) were obtained from three locations posterior to the bregma at approximately 0.2, 0.7, and 1.2 mm (Paxinos and Watson, 2006). Ten randomly selected areas (160 μm × 160 μm per field) were chosen in the lesion core of each section, and images of GRP78 and RECA1 immunoreactivity were then obtained from these areas at ×400 magnification under constant viewing conditions. Three days after 3-NP injection, two areas of the lesion core (the core-periphery and core-epicenter) were clearly distinguishable in the experimental rats on the basis of the presence of GRP78-positive cells; therefore, 10 areas were chosen from each of the two areas. The area covered by GRP78 and RECA-1 was estimated, and GRP78 coverage was expressed as a percentage of the total RECA1-positive vascular area. Differences in staining intensity between groups were assessed with one-way analysis of variance (ANOVA) followed by *post hoc* Bonferroni tests for multiple comparisons. Differences with *P*-values less than 0.05 were considered statistically significant. All statistical analysis was performed using GraphPad Prism version 5 (GraphPad Software Inc., San Diego, CA, United States).

In addition, to quantify the time-dependent changes in GRP78-positive microglia/macrophages in the striatum in rats treated with 3-NP, sections obtained from control and experimental rats (from the three locations described above) at 1, 3, and 7 days after 3-NP injection (*n* = 3 per time point) were double-labeled for GRP78 and Iba1. Ten areas (160 μm × 160 μm per field) were chosen in the lesion core of each section and the corresponding striatum from control sections, and GRP78/Iba1 double-labeled cells were counted only when their nuclei could be clearly observed. The cell counts are presented as the mean ± standard error of the mean (SEM). Data analysis was performed as described above.

### Assessment of Blood–Brain Barrier (BBB) Leakage

To detect BBB leakage in 3-NP-injected rats, experimental rats (*n* = 3 animals) were deeply anesthetized on days 3 and 7 after 3-NP injection and intravenously administered fluorescein isothiocyanate (FITC)-albumin (2 mg diluted in 0.1 ml saline; Sigma-Aldrich) via the tail vein. One hour after injection, animals were deeply anesthetized and perfused transcardially with fixative, as described above. Coronal cryostat sections (25-μm-thick) from animals injected with FITC-albumin were incubated overnight at 4°C with a mixture of rabbit polyclonal antibody against GRP78 (1:2000; Abcam) and one of following antibodies: goat polyclonal antibody against Iba1 (1:500; Abcam), mouse monoclonal antibody against RECA1 (1:200; Bio-Rad), or mouse monoclonal antibodies against GFAP (1:700; Millipore). This was followed by 2-h incubation with appropriate secondary antibodies as follows: Cy3-conjugated donkey anti-goat antibody (1:2000; Jackson ImmunoResearch), Cy3-conjugated goat anti-mouse antibody (1:2000; Jackson ImmunoResearch), or Alexa Fluor 647-tagged donkey anti-rabbit antibody (1:300; Thermo Fisher). Counterstaining of cell nuclei was carried out using DAPI for 10 min.

### Immunoelectron Microscopy

For the correlative light- and electron-microscopic study, vibratome sections (300-μm-thick) from experimental rats at 3 days after 3-NP injection were cryoprotected with 2.3 M sucrose in 0.1 M PB and frozen in liquid nitrogen. Semi-thin cryosections (1-μm-thick) were cut at −100°C with a glass knife in a Leica EM UC7 ultramicrotome equipped with an FC7 cryochamber (Leica). The sections were double-labeled at 4°C overnight using a mix of rabbit polyclonal antibody against GRP78 (1:2000; Abcam) and one of following antibodies: goat polyclonal antibody against Iba1 (1:500; Abcam), mouse monoclonal antibody against RECA1 (1:200; Bio-Rad), or mouse monoclonal antibody to nestin (1:500; Millipore). Antibody staining was visualized using Alexa Fluor 488-FluoroNanogold-anti-rabbit Fab’ (1:300; Nanoprobes; Yaphank, NY, United States) and either Cy3-conjugated goat anti-mouse antibody (1:2000, Jackson ImmunoResearch) or Alexa Fluor 647-tagged donkey anti-goat antibody (1:300; Thermo Fisher). Sections were counterstained with DAPI for 10 min. Coverslipped sections were examined with a confocal microscope and photographed at various magnifications with a differential interference contrast setting to find specific areas for later examination by electron microscopy. After the coverslips had been floated off the sections, silver enhancement was performed using the HQ silver enhancement kit (Nanoprobes) for 3 min, and the tissues were prepared further for electron microscopy. After post-fixation, dehydration, and embedding in Epon 812 (Polysciences, Warrington, PA, United States), areas of interest were excised and glued onto resin blocks. After being cut into ultrathin sections 70–90 nm thick, they were observed in an electron microscope (JEM 1010; JEOL, Tokyo, Japan) with slight uranyl acetate staining.

## Results

### Spatial and Temporal Expression of GRP78 in the Striatum of Rats Treated With 3-NP

Consistent with our previous data (Choi et al., 2017; Riew et al., 2017; Jin et al., 2018b), approximately 70% of rats treated with 3-NP in our study developed characteristic neurological deficits including hindlimb impairment, recumbency, and impaired postural adjustments. In addition, all the animals examined in this study showed comparable expression patterns of GRP78 at each time point after 3-NP injection.

**FIGURE 1:**
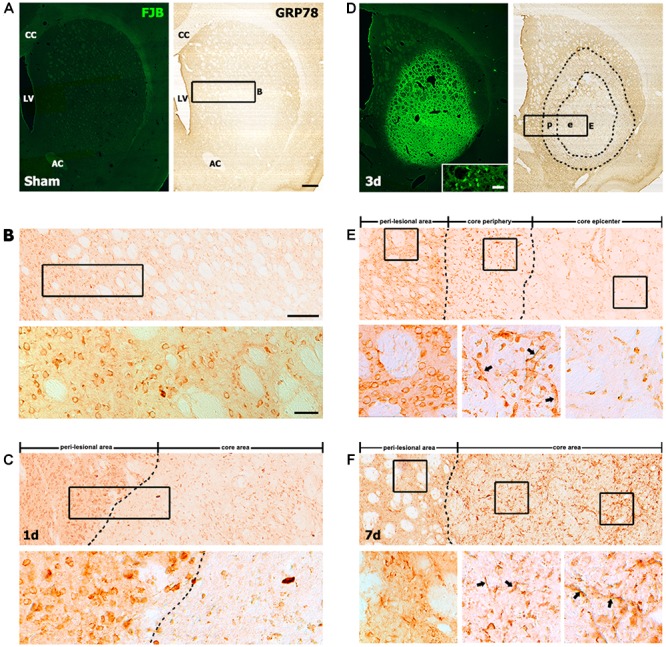
Representative images showing the temporal expression of the 78-kDa glucose-regulated protein (GRP78) in striatal sections from saline-treated control and 3-nitropropionic acid-injected rats. **(A,B)** Lower- **(A)** and higher- **(B)** magnification views of coronal sections from saline-treated controls. **(A)** Serial striatal sections stained with Fluoro-Jade B (FJB) and anti-GRP78 antibody, showing no specific staining for FJB in the striatum. **(B)** Upper and lower panels of B are higher magnification images of the boxed area of A and upper panel of B, respectively, showing that GRP78 is present in neuron-like cells. **(C)** At day 1 after lesion induction, a spatial difference can be observed between GRP78 expression in the lesion core (right side of the broken line) and that in the peri-lesional area. The boxed area in upper panel is enlarged in the lower panel, showing that most neuronal profiles are absent in the lesion core, while intense GRP78 immunoreactivity is still evident in neurons in the peri-lesional area. **(D)** At 3 days after lesion induction, the core of the lesion, which is clearly distinguished by intense FJB staining, can be divided into two distinct areas on the basis of GRP78 immunoreactivity: the epicenter (e) and the lesion periphery (p); the border between these areas is indicated by the broken line. (Inset in left panel of **D**) Higher magnification image of an FJB-stained section, showing intense FJB staining within neuronal profiles in the lesion core. (**E**, Upper) Higher magnification image of the boxed area of **D**, showing the distinct expression patterns of GRP78 in the perilesional area, lesion periphery, and epicenter. Boxed areas in these three parts are enlarged in the corresponding lower panels of **E**. (**E**, Lower) Intense GRP78 expression is observed in vascular profiles (arrows in middle panel) and cells with round cell bodies in the lesion periphery, while no prominent GRP78 expression is visible in the lesion epicenter. (**F**, Upper) At day 7 after lesion induction, GRP78 expression is more densely distributed throughout the lesion core (right side of the broken line). (**F**, Lower) Higher magnification images of boxed areas in the three areas, i.e., the perilesional area, lesion periphery, and epicenter, from the corresponding images in the upper panel. Note that vessel-associated GRP78 immunoreactivity is evident in both the lesion periphery (arrows in middle panel) and the epicenter (arrows in right panel). CC, corpus callosum; LV, lateral ventricle; AC, anterior commissure. Scale bars represent 500 μm for **A**, **D**; 200 μm for upper panels in **B**, **C**, **E**, **F**; and 50 μm for the inset in the left panel in **D** and lower panels in **B**, **C**, **E**, **F**.

We first performed immunohistochemistry to examine the spatiotemporal distribution of GRP78-positive cells in the striata of rats treated with 3-NP. In the striata of saline-treated controls, we observed no specific staining for FJB, which labels degenerating neurons, and GRP78 immunoreactivity was observed in neuron-like cells (Figures 1A,B), as reported previously (Jin et al., 2018a). One day after the last 3-NP injection, a well-demarcated lesion core was evident in the lateral part of the striatum. GRP78-positive neurons had mostly disappeared in the lesion core, while neuronal GRP78 expression persisted in the peri-lesional area (Figure 1C). Three days after lesion induction, the lesion core, which was characterized by intense FJB staining, could be clearly divided into two areas according to the GRP78 expression profile: the epicenter and periphery of the lesion core (Figures 1D,E). A higher magnification image of the lesion core revealed that neuronal profiles were positive for the neuronal degeneration marker FJB (inset in Figure 1D). In the periphery of the lesion core, intense GRP78 immunoreactivity was observed in vascular profiles and cells with round cell bodies, while only weak GRP78 immunoreactivity was observed in the lesion epicenter. At day 7 after lesion induction, GRP78 immunoreactivity was evenly distributed throughout the lesion core, including the periphery and the center (Figure 1F). This expression pattern was maintained on day 14 (data not shown).

### Characterization of GRP78-Positive Cells in the Striatum After 3-NP Injection

**FIGURE 2:**
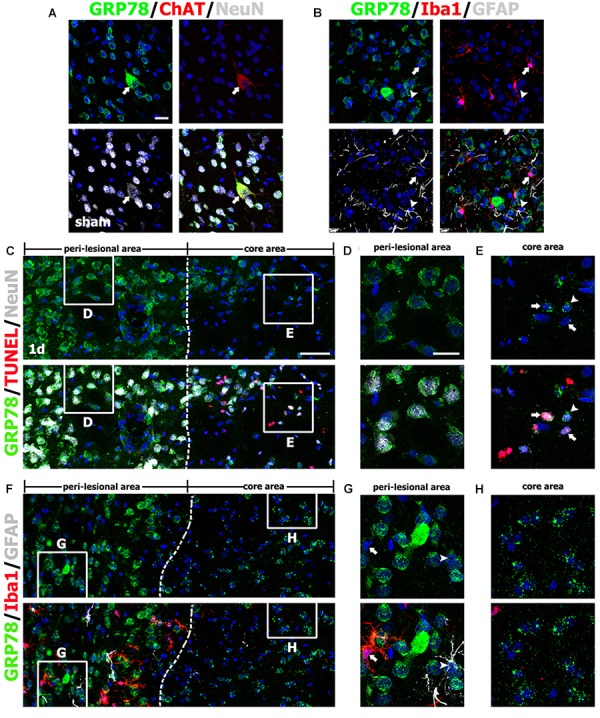
Characterization of glucose-regulated protein (GRP78)-positive cells in the striata of saline-treated control and 3-nitropropionic acid-injected rats on day 1. **(A)** Triple-labeling of GRP78, neuronal nuclear protein (NeuN), and choline acetyltransferase (ChAT), a specific marker for cholinergic neurons, in control striatum, showing that GRP78 expression is localized in almost all NeuN-positive neurons and also in some cholinergic interneurons (arrows). **(B)** Triple-labeling of GRP78, ionized calcium-binding adaptor molecule 1 (Iba1), and glial fibrillary acidic protein (GFAP) in the striatum of a control rat, showing that neither microglia (arrows) nor astrocytes (arrowheads) show specific immunoreactivity for GRP78. **(C–E)** Lower- **(C)** and higher- **(D,E)** magnification views of sections triple-labeled for GRP78, NeuN, and terminal deoxynucleotidyl transferase dUTP nick end labeling (TUNEL) 1 day after lesion induction. The boxed areas of the peri-lesional area (left side of the broken line) and the lesion core in **C** are enlarged in **D** and **E**, respectively. Notably, GRP78 expression is very weak to negligible in nearly all TUNEL-positive (arrows in **E**) and TUNEL-negative (arrowheads in **E**) neurons in the lesion core, while neurons in the peri-lesional area show prominent GRP78 immunoreactivity. **(F–H)** Lower- **(F)** and higher- **(G,H)** magnification views of section triple-labeled for GRP78, Iba1, and GFAP 1 day after lesion induction. The boxed areas of the peri-lesional area (left side of the broken line) and the lesion core in **F** are enlarged in **G** and **H**, respectively. Note that astrocytes (arrowheads in **G**) and microglia (arrows in **G**) in the peri-lesional area show very weak immunoreactivity for GRP78. Cell nuclei are stained with 4′,6-diamidino-2-phenylindole. Scale bars represent 50 μm for **C**, **F**; and 20 μm for **A**, **B**, **D**, **E**, **G**, **H**.

As described above, the pattern of GRP78 immunoreactivity in the lesion core changed during the post-injury period. Therefore, to clarify the phenotypes of GRP78-positive cells in the striata from control and experimental rats, we performed triple-labeling with GRP78 and cell type-specific markers. In the striata from control rats, GRP78 immunoreactivity was evident in almost all striatal neurons expressing NeuN, including large cholinergic interneurons expressing choline acetyltransferase, a specific marker for cholinergic neurons (Figure 2A), but not in astrocytes or microglia (Figure 2B), consistent with the findings of our previous study (Jin et al., 2018a).

One day after lesion induction, most GRP78-positive neuronal profiles were absent from the lesion core, which was confirmed by triple-labeling of GRP78, NeuN, and TUNEL (Figure 2C). Striatal neurons in the lesion core, most of which were positive for TUNEL, were devoid of significant GRP78 immunoreactivity (Figure 2E), while neurons in the peri-lesional area showed evident GRP78 immunoreactivity (Figure 2D). At this time point, triple-labeling of GRP78, Iba1, and GFAP revealed that the lesion core was clearly demarcated by the absence of astrocytes and microglia with normal morphology, while both glial cell types were present in the peri-lesional area (Figures 2F,H). Observation of the peri-lesional area at higher magnification revealed weak GRP78 immunoreactivity in astrocytes and microglia, both of which had not yet shown typical reactive phenotypes (Figure 2G).

**FIGURE 3:**
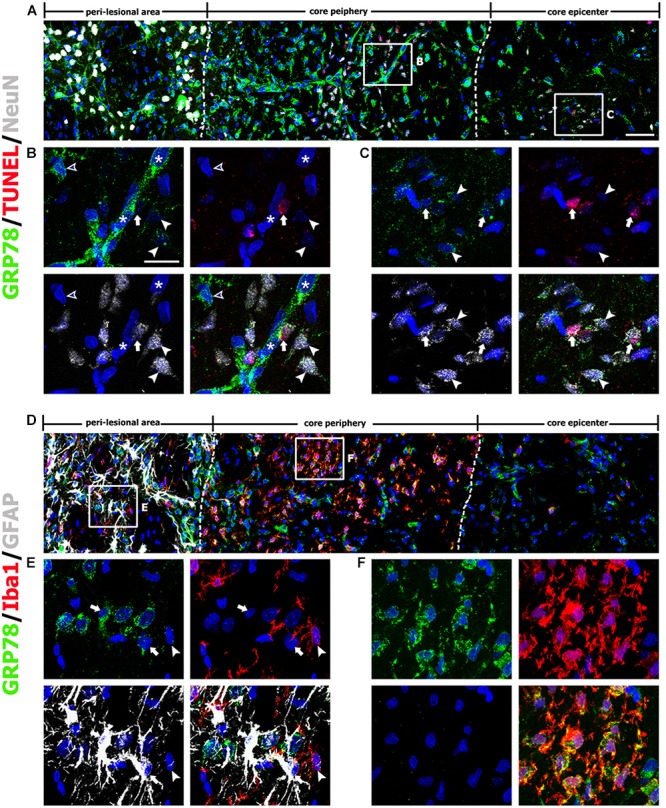
Characterization of glucose-regulated protein (GRP78)-positive cells in lesioned striatum on day 3 after 3-nitropropionic acid injection. **(A)** Triple-labeling of GRP78, NeuN, and terminal deoxynucleotidyl transferase dUTP nick end labeling (TUNEL) showing that neuronal GRP78 expression is evident in the perilesional area, while GRP78 is virtually absent in dying or dead neurons in the lesion core. The two broken lines indicate the borders of three areas: the peri-lesional area, lesion periphery, and epicenter. The corresponding boxed areas in the lesion periphery and epicenter in **A** are enlarged in **B**, **C**. **(B,C)** In both the lesion periphery **(B)** and the epicenter **(C)**, both TUNEL-positive (arrows) and TUNEL-negative (arrowheads) neurons show negligible immunoreactivity. Notably, in the lesion periphery, intense GRP78 expression is localized within the vascular profiles (asterisks in **B**) and presumptive activated microglia (open arrowheads in **B**), while no specific GRP78 expression is detectable in the epicenter. **(D)** Triple-labeling of GRP78, Iba1, and GFAP showing that the lesion core can be divided into two areas: the core periphery, which is heavily infiltrated by activated microglia, and the epicenter, which is devoid of Iba1-positive microglia. Notably, GFAP immunoreactivity is absent in both the periphery and the epicenter. **(E,F)** Higher-magnification views of the corresponding boxed areas in **D**. **(E)** In the peri-lesional area, GRP78 expression can be observed in reactive astrocytes (arrows) and activated microglia (arrowheads). **(F)** In the lesion periphery, intense GRP78 expression is visible in almost all Iba1-positive cells. Cell nuclei are stained with 4′,6-diamidino-2-phenylindole. Scale bars represent 50 μm for **A D**; and 20 μm for **B**, **C**, **E**, **F**.

On day 3 after lesion induction, triple-labeling of GRP78, NeuN, and TUNEL revealed that specific GRP78 immunoreactivity was virtually absent in dying or dead neurons in the lesion core (Figure 3A). As mentioned above, the lesion core could be divided into two areas: the lesion periphery, in which intense GRP78 expression was localized within the vascular profiles and cells with round cell bodies (Figure 3B), and the epicenter, which was devoid of specific GRP78 expression (Figure 3C). This finding was further supported by triple-labeling of GRP78, Iba1, and GFAP; in the lesion core that was devoid of GFAP immunoreactivity, GRP78 and Iba1 had overlapping regional distributions confined to the lesion periphery, while no Iba1-positive microglia were observed within the epicenter of the lesion core (Figures 3D,F). By contrast, GRP78 immunoreactivity was more evident in reactive astrocytes and activated microglia in the peri-lesional area on day 3 when compared to GRP78 immunoreactivity at day 1 after lesion induction (Figure 3E).

**FIGURE 4:**
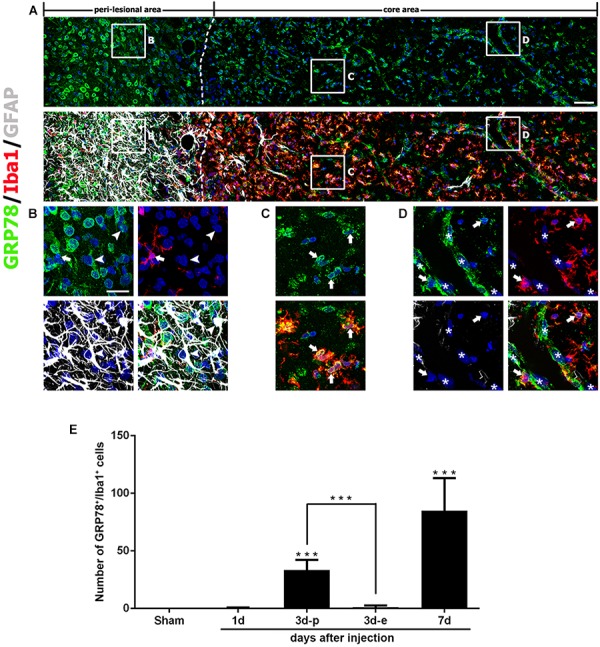
Characterization of glucose-regulated protein (GRP78)-positive cells in lesioned striatum on day 7 after 3-nitropropionic acid injection. **(A)** Triple-labeling of GRP78, Iba1, and GFAP showing that the lesion core (right side of the broken line) is devoid of GFAP immunoreactivity, but is evenly filled with Iba1-positive cells, nearly all of which express GRP78. The boxed areas in the peri-lesional area and the lesion core are enlarged in the corresponding panels **B–D**. **(B)** In the peri-lesional area, evident GRP78 immunoreactivity is observed in reactive astrocytes (arrowheads) and activated microglia (arrows). **(C,D)** In the lesion core, intense GRP78 expression is observed in the vascular profiles (asterisks) and in activated microglia/macrophages (arrows). **(E)** Quantitative temporal analysis showing that the number of GRP78-positive microglia/macrophages increases progressively over a 7-day period after lesion induction. Note that the number of GRP78-positive microglia/macrophages is significantly higher in the lesion periphery than in the epicenter on day 3. The data are expressed as the mean ± standard error of the mean. ^∗∗∗^*P* < 0.001 vs. saline-treated controls. Cell nuclei are stained with 4′,6-diamidino-2-phenylindole. Scale bars represent 50 μm for **A**; and 20 μm for **B–D**.

Seven days after lesion induction, triple-labeling of GRP78, Iba1, and GFAP revealed that the lesion core was evenly populated with Iba1-positive cells, nearly all of which expressed GRP78, although GRP78 expression was also observed in the vascular profiles (Figures 4A,C,D). In the peri-lesional area, intense GRP78 expression was observed in astrocytes and microglia, both of which showed distinct reactive phenotypes (Figure 4B).

As described above, GRP78 expression was induced in almost all Iba1-positive cells, including activated microglia/macrophages in the lesion core, and they appeared to gradually increase in number over 7 days after lesion induction. Quantitative temporal analysis revealed a progressive increase in the number of GRP78-positive microglia/macrophages at 3–7 days post-lesion (Figure 4E). In particular, the number of GRP78-positive microglia/macrophages in the lesion periphery was significantly higher than that in the epicenter in the striatum 3 days post-lesion.

### Induction of GRP78 Expression Within Vascular Structures in the Striatum After 3-NP Injection

**FIGURE 5:**
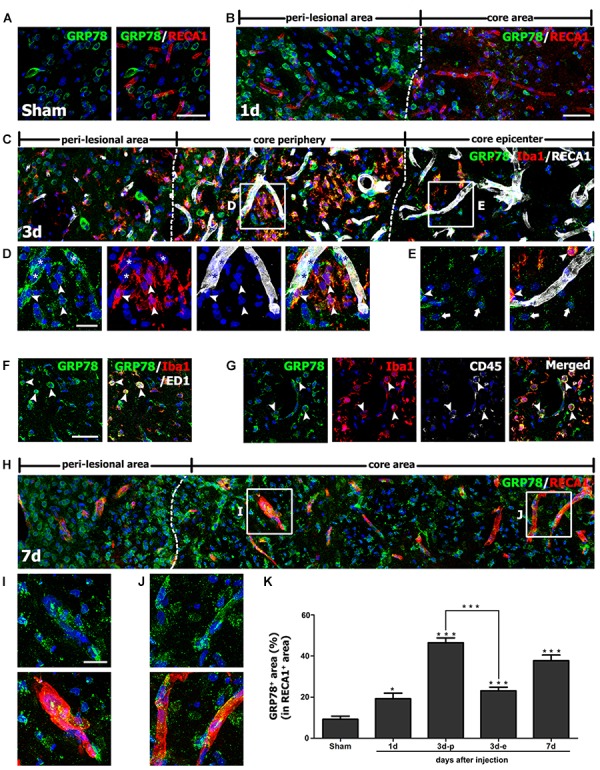
Characterization of glucose-regulated protein (GRP78) expression associated with the vasculature in control and lesioned striata after 3-nitropropionic acid injection. **(A)** Double-labeling of GRP78 and the endothelial cell marker RECA1 in control striatum, showing that GRP78 immunoreactivity is negligible or absent within vessels. **(B)** Double-labeling of GRP78 and RECA1 1 day after lesion induction, showing that GRP78 immunoreactivity within vessels is negligible or very weak in the lesion core (right side of the broken line). **(C–E)** Lower- **(C)** and higher- **(D,E)** magnification views of sections triple-labeled for GRP78, Iba1, and RECA1 3 days after lesion induction. The boxed areas of the lesion periphery and the epicenter in **C** are enlarged in **D** and **E**, respectively. Notably, the lesion periphery is heavily infiltrated by GRP78/Iba1 double-labeled microglia/macrophages (arrowheads in **D**), while only some Iba1-positive microglia/macrophages can be detected in the epicenter (arrowheads in **E**). In addition, GRP78 expression is detectable in association with most of the vasculature (asterisks in **D**) in the lesion periphery. Arrows in **E** indicate presumptive dying or dead neurons that are devoid of significant GRP78 expression. **(F,G)** Triple-labeling of GRP78, Iba1, and either ED1 or CD45 on day 3 after lesion induction, showing that GRP78 is expressed in nearly all of ED1- (arrowheads in **F**) or CD45-positive cells (arrowheads in **G**), corresponding to only a small fraction of the GRP78/Iba1 double-labeled cells. Notably, these triple-labeled cells are frequently associated with blood vessels. **(H)** Double-labeling of GRP78 and RECA1 at 7 days after lesion induction, showing that GRP78 expression is evenly distributed throughout the lesion core (right side of the broken line). **(I,J)** Higher magnification images of the boxed areas in **H**, showing that GRP78 immunoreactivity is localized to nearly all vessels in both the lesion periphery **(I)** and epicenter **(J)**. **(K)** Quantitative temporal analysis of the proportion of vascular areas occupied by GRP78 immunoreactivity, within all RECA1-positive vessels. This proportion increases progressively by day 1, and then decreases slightly on day 7. Note that the vascular area covered by GRP78 is significantly higher in the lesion periphery than in the epicenter on day 3. The data are expressed as the mean ± standard error of the mean. ^∗^*P* < 0.05 and ^∗∗∗^*P* < 0.001 vs. saline-treated controls. Cell nuclei are stained with 4′,6-diamidino-2-phenylindole. Scale bars represent 50 μm for **A–C**, **F–H**; and 20 μm for **D**, **E**, **I**, **J**.

The above findings prompted us to investigate the association between GRP78 and blood vessels. Therefore, we performed double-labeling of GRP78 and the vascular endothelial cell marker RECA1. In striata from control rats (Figure 5A) and rats after 1 day of lesion induction (Figure 5B), GRP78 expression was absent or very weak within vascular profiles. At 3 days after lesion induction, triple-labeling of GRP78, RECA1, and Iba1 revealed that vessel-associated GRP78 expression was prominent in the lesion periphery, where GRP78-positive activated microglia/macrophages had accumulated (Figures 5C,D). In contrast, GRP78 immunoreactivity was observed only in a small fraction of the vasculature in the lesion epicenter, where microglia had not yet infiltrated (Figure 5E). We further characterized these microglia/macrophages expressing GRP78 by triple-labeling of GRP78, Iba1, and either ED1, which is expressed in the membranes of phagolysosomes in microglia and macrophages (Damoiseaux et al., 1994), or CD45, which is expressed in all nucleated hematopoietic cells or leukocytes (Penninger et al., 2001). As shown in Figures 5F,G, a small fraction of the GRP78/Iba1 double-labeled cells in the lesion core expressed ED1 or CD45 and showed amoeboid macrophage-like phenotypes, indicating that they were likely to be blood-derived macrophages, as reported previously (Guillemin and Brew, 2004).

Seven days after lesion induction, GRP78 immunoreactivity was localized to nearly all vessels in both the epicenter and the periphery of the lesion (Figures 5H–J). Next, we determined the relative proportion of vascular profiles occupied by GRP78 among all RECA1-positive vessels in the lesion core over a 7-day period after lesion induction. As shown in Figure 5K, 9.2% and 19.2% of all RECA1-positive vascular areas were covered by areas of GRP78 immunoreactivity in control sections and in lesioned striata 1 day after lesion induction, respectively. At 3 days after lesion induction, this proportion in the lesion periphery rose to 46.5%, which was significantly higher than that in the epicenter (23.1% of all vessels). At 7 days after lesion induction, the GRP78-positive area comprised 37.8% of the vascular area in both the periphery and the epicenter of the lesion core.

### Spatiotemporal Coincidence Between Vascular GRP78 Expression and BBB Leakage in the Lesion Core After 3-NP Injection

**FIGURE 6:**
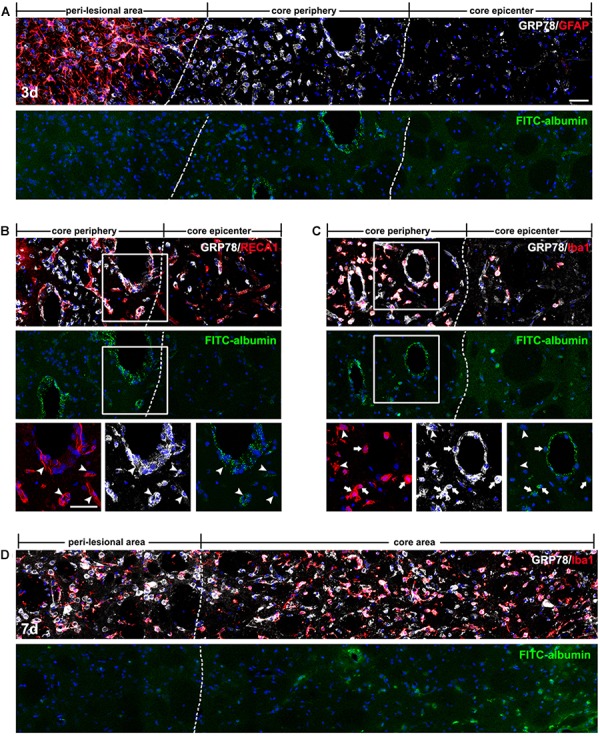
Spatiotemporal coincidence between vascular glucose-regulated protein (GRP78) expression and blood–brain barrier (BBB) leakage, as indicated by extravasation of fluorescein isothiocyanate (FITC)-albumin in the striata lesion by 3-nitropropionic acid injection. **(A)** Triple-labeling of FITC-albumin, GRP78, and GFAP 3 days after lesion induction, showing that vessels labeled with FITC-albumin are distributed only in the periphery of the lesion core, and not in the peri-lesional area or the lesion epicenter. The two broken lines indicate the borders of the three areas: the peri-lesional area, lesion periphery, and epicenter. **(B)** Triple-labeling of FITC-albumin, GRP78, and RECA1 at day 3, showing that both FITC-albumin and GRP78 are detectable in nearly identical vessels (arrowheads) in the lesion periphery. **(C**) Triple-labeling of FITC-albumin, GRP78, and Iba1 at day 3, showing that in addition to vascular walls, FITC-albumin is detected in amoeboid-like brain macrophages (arrows), but not in activated microglia with evident processes (arrowheads). **(D)** Triple-labeling of FITC-albumin, GRP78, and Iba1 at 7 days after lesion induction, showing that tracer extravasation is detectable throughout the lesion core (right side of the broken line). Cell nuclei are stained with 4′,6-diamidino-2-phenylindole. Scale bars represent 50 μm for **A–D**.

In order to determine whether vessel-associated GRP78 expression could be related to the impairment of the BBB that occurred in the striatum of 3-NP treated rats, we infused FITC-albumin into the circulation via the tail vein at days 3 and 7 after 3-NP injection, and allowed the tracer to circulate for 1 h. On day 3 after lesion induction, triple-labeling of FITC-albumin, GRP78, and GFAP revealed that vessels with FITC-albumin were exclusively confined to the lesion core, where GFAP immunoreactivity was absent, while no noticeable vascular fluorescence was observed in the peri-lesional area (Figure 6A). In particular, we found that FITC-labeled vessels were more apparent in the lesion periphery than in the epicenter. This observation was supported by the results of triple-labeling of GRP78, FITC-albumin, and RECA1, which showed that both FITC-albumin and GRP78 were co-expressed in almost all the same vessels in the lesion periphery (Figure 6B). In addition, triple-labeling of FITC-albumin, GRP78, and Iba1 showed that FITC-labeled vessels were more prominent in the lesion periphery, where GRP78-positive microglia/macrophages had infiltrated, than in the microglia-free epicenter (Figure 6C). Higher magnification revealed that, in close proximity to FITC-labeled vessels, FITC-albumin accumulated within amoeboid-like brain macrophages, but not in activated microglia with evident processes, indicating that leaked FITC-albumin could be sequestered by brain macrophages (Figure 6C). At 7 days after lesion induction, triple-labeling of GRP78, FITC-albumin, and Iba1 revealed that FITC-albumin could be detected in both the periphery and the epicenter of the lesion core, indicating the overlapping distribution of GRP78-positive microglia/macrophage and BBB leakage (Figure 6D).

### Phenotypic Characterization of Vessel-Associated GRP78-Positive Cells in the Lesion Core After 3-NP Injection

**FIGURE 7:**
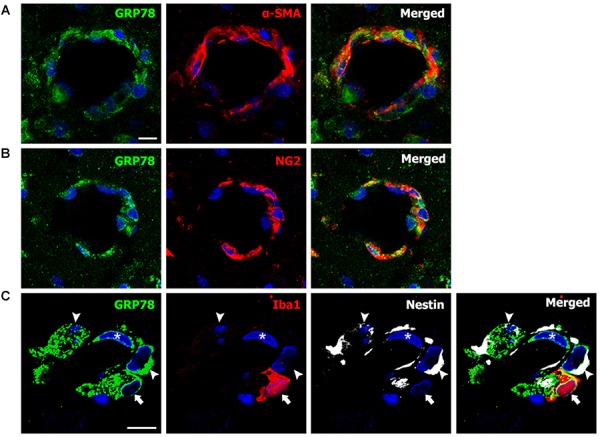
Phenotypic characterization of vessel-associated glucose-regulated protein (GRP78)-positive cells in the lesion periphery on day 3 after lesion induction by 3-nitropropionic acid injection. **(A,B)** Double-labeling of GRP78 and two smooth muscle cell markers, alpha-smooth muscle actin (α-SMA) and NG2, showing that prominent GRP78 immunoreactivity is visible in smooth muscle cells. **(C)** Triple-labeling of GRP78, Iba1, and nestin showing that GRP78 expression can be detected in endothelial cells (asterisks), Iba1-positive microglia/macrophages (arrows), and nestin-positive perivascular cells (arrowheads). Cell nuclei are stained with 4′,6-diamidino-2-phenylindole. Scale bars represent 10 μm for **A–C**.

For the phenotypic characterization of GRP78-expressing cells associated with the vasculature in the lesion core, we performed double-labeling using GRP78 and two smooth muscle cell markers, α-SMA or NG2, both of which label smooth muscle cells (Jin et al., 2018b). As shown in Figures 7A,B, GRP78-positive cells in vascular profiles were positive for both smooth muscle cell markers. Next, we performed triple-labeling of GRP78, Iba1, and nestin, because a subset of vascular wall cells that are distinct from endothelial cells, pericytes, or smooth muscle cells express nestin, and can transform into fibroblast-like cells in the ischemic brain (Shin et al., 2013). As shown in Figure 7C, GRP78 expression was clearly detected in three types of vasculature-associated cells: endothelial cells, Iba1-positive microglia/macrophages, and nestin-positive perivascular cells that were devoid of Iba1 immunoreactivity. Thus, our data indicate a phenotypic heterogeneity of vascular GRP78-positive cells in the striatal lesions.

**FIGURE 8:**
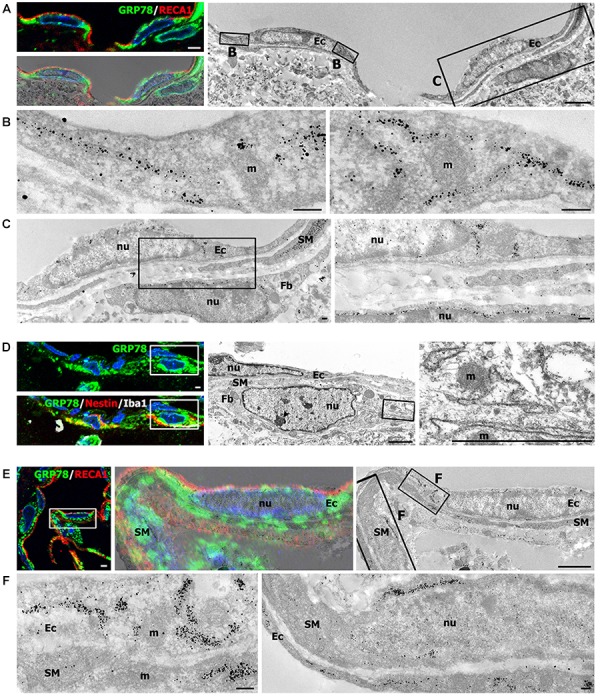
Ultrastructural characterization of vascular glucose-regulated protein (GRP78)-positive cells in the lesion periphery at 3 days after lesion induction by 3-nitropropionic acid injection. **(A)** Confocal microscopic image of a semi-thin section double-labeled with GRP78 and RECA1 (left upper panel), image of confocal microscopic data overlaid onto the corresponding electron microscopic image (left lower panel), and the corresponding transmission electron microscopic image obtained from the same field (right panel). **(B,C)** Higher-magnification views of the boxed areas in **A**. The boxed area in the left panel of **C** is enlarged in the right panel of **C**. Notably, GRP78 protein, as indicated by silver-enhanced immunogold particles, is specifically localized to the cisternae of the rough endoplasmic reticulum (rER), but not to other organelles within endothelial cells (Ec), such as mitochondria (m) or nuclei (nu). In addition, GRP78 protein is detectable in perivascular cells (Fb) that are closely apposed to the outer part of endothelial cells and smooth muscle cells (SM). **(D)** Confocal microscopic image of a semi-thin section triple-labeled for GRP78, Iba1, and nestin (left panel), and the corresponding electron microscopic image of the boxed areas in the left panels (middle panel). The boxed area in the middle panel is enlarged in the right panel. Note that the GRP78/nestin double-labeled cell (Fb) has a large euchromatic nucleus (nu) with prominent nucleolus (arrowhead), and was invariably located outside the endothelial cells (Ec) and smooth muscle cells (SM). Furthermore, GRP78 protein is mainly localized in the rER/perinuclear cisternae, but not in the mitochondria (m) or nucleus (nu) of GRP78/nestin double-labeled cells. **(E)** Confocal microscopic image of a semi-thin section double-labeled for GRP78 and RECA1 (left panel), the corresponding transmission electron microscopic image of the boxed area in the left panel (right panel), and their overlay image (middle panel). **(F)** Higher-magnification images of the boxed areas in the right panel of **E**. Notably, the silver-enhanced immunogold particles targeted to GRP78 are specifically associated with the perinuclear cistern and the cisternae of the rER of endothelial cells (Ec) and smooth muscle cells (SM), but not with mitochondria (m). Cell nuclei are stained with 4′,6-diamidino-2-phenylindole. Scale bars represent 2 μm for **A**, **D**, **E**; 1 μm for **C**; and 0.2 μm for **B**, **F**.

Light microscopic imaging alone was not sufficient to clarify the identity of vascular GRP78-positive cells and to establish the precise subcellular localization of GRP78 protein within these cells. Therefore, we performed correlative light- and immunogold-electron microscopic imaging to determine the precise localization of the light microscopic signals from subcellular structures. First, semi-thin sections double-labeled for GRP78 and RECA1 were observed using confocal microscopy, and the same semi-thin sections were subsequently subjected to electron microscopy (Figures 8A–C,E,F). Overlay of the confocal microscopy and transmission electron microscopy data confirmed that GRP78 protein, as indicated by silver-enhanced immunogold particles, was specifically localized to the cisternae of the rough ER in endothelial cells, but not to other organelles such as mitochondria (Figures 8B,C,F). In addition, GRP78 protein was detected in cells that were closely apposed to the outer part of endothelial cells and smooth muscle cells (Figures 8A,C), indicating that these GRP78-expressing cells may correspond to nestin-positive cells, based on their location in the perivascular space. Thus, semi-thin sections triple-labeled for GRP78, nestin, and Iba1 were further analyzed with electron microscopy. As shown in Figure 8D, GRP78/nestin double-labeled cells that were devoid of Iba1 labeling were characterized by a large euchromatic nucleus with a prominent nucleolus and invariably lay outside the endothelial and smooth muscle cells, indicating that they may correspond to perivascular fibroblast-like cells, as described previously (Shin et al., 2013). GRP78 protein in these cells was mainly localized in the rough ER/perinuclear cisternae, some of which showed marked dilatation (Figures 8C,D). In smooth muscle cells, silver-enhanced immunogold particles targeted to GRP78 were specifically associated with the perinuclear cistern and the cisternae of the rough ER (Figures 8E,F).

## Discussion

**FIGURE 9:**
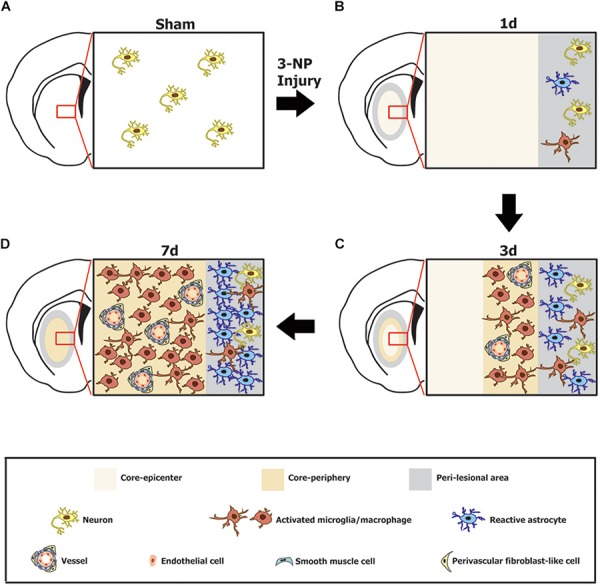
Schematic representation of the phenotypic heterogeneity of GRP78-positive cells in the lesion core and perilesional area in the striata after lesion induction using 3-nitropropionic acid injection. **(A)** In the saline-treated control striatum, GRP78 immunoreactivity is almost exclusively localized to striatal neurons. **(B)** One day after 3-NP injection, neuronal profiles are absent from the lesion core, while neurons in the peri-lesional area show evident GRP78 immunoreactivity. In addition, astrocytes and microglia in the peri-lesional area show very weak immunoreactivity for GRP78. **(C)** Three days after lesion induction, GRP78 expression is induced in association with activated microglia/macrophages and the vascular wall, including endothelial cells, smooth muscle cells, and adventitial fibroblast-like cells in the lesion periphery, although these cell types are absent from the lesion epicenter. In addition, evident GRP78 immunoreactivity is observed in reactive astrocytes and activated microglia in the perilesional area. **(D)** Seven days after lesion induction, intense GRP78 expression is observed in the vascular profiles and in activated microglia/macrophages throughout the lesion core. In addition, intense GRP78 expression is observed in reactive astrocytes and activated microglia in the peri-lesional area.

We recently reported that, in a rat model of stroke, GRP78 expression is induced in activated glial cells, predominantly in brain macrophages and reactive astrocytes in the infarct and peri-infarct areas, respectively (Jin et al., 2018a). In the present study, by using a 3-NP injection model, we further investigated the induction of GRP78 expression in response to neurotoxic insults. In agreement with our previous findings (Jin et al., 2018a), constitutive GRP78 expression was observed in striatal neurons, and expression was induced in association with activated glial cells in the striata of rats treated with 3-NP. Glial induction of prominent GRP78 expression could be attributed to activated microglia/macrophages and reactive astrocytes. In addition, the present study provides new evidence for the prominent induction of GRP78 within vascular profiles in the striatal lesion. Although the model of ischemic stroke and the 3-NP model have commonalities including BBB breakdown and lead to the formation of striatal lesions consisting of a lesion core and peri-lesional area (Mu et al., 2016), the 3-NP model employed in the present study shows a better-preserved lesion core, because well-demarcated striatal lesions do not spread to the cerebral cortex (Duran-Vilaregut et al., 2010; Mu et al., 2016; Riew et al., 2017). Thus, this 3-NP model allows a detailed characterization of the time course of GRP78 expression in the lesion core, especially within vascular profiles that are difficult to observe when tissue damage is severe. Together with our previous findings (Jin et al., 2018a), our data demonstrate a strong correlation between GRP78 expression and an activated functional status of neuroglial cells during CNS insults, and provide novel evidence to show that cells of the vascular wall also contribute to the increased GRP78 expression in the lesion core (Figure 9).

Notably, we found that vascular induction of GRP78 showed characteristic spatiotemporal patterns during the post-injury period. GRP78 expression was negligible or very weak in the control striata and at 1 day after lesion induction. However, expression was detectable at the edge of the lesion core by day 3 after lesion induction, and was evenly distributed throughout the lesion core by day 7. The proportion of vascular area covered by GRP78 immunoreactivity within all RECA1-positive vessels was significantly higher in the lesion periphery than in the epicenter 3 days after lesion induction. Interestingly, vascular GRP78 expression was correlated temporally and spatially with infiltration of activated microglia into the lesion core. In agreement with our previous reports (Jin et al., 2018b), activated microglia/macrophages appeared at the lesion edge 3 days after lesion induction, with infiltration throughout the lesion core being clearly observed by day 7. This distribution of reactive microglia is analogous with previously published data on their band-like accumulation in regions surrounding the ischemic core during the first week of ischemic stroke (Li et al., 2013, 2014), indicating that instead of containing pre-existing microglia, the lesion core was progressively infiltrated by microglia migrating from the peri-lesional area. These activated microglia/macrophages showed intense immunoreactivity for GRP78, as previously reported (Fan et al., 2015; Yang et al., 2015; Corsetti et al., 2017; Jin et al., 2018a). Thus, the temporal patterns of vascular GRP78 appeared to overlap with the period during which GRP78 was expressed by microglia/macrophages recruited into the lesion core. Recent studies have shown that under pathological conditions such as ischemic insults, BBB disruption is closely correlated with accumulation of activated microglia, suggesting that microglia are associated with blood vessel dysfunction and pro-inflammatory signaling (Matsumoto et al., 2012; Barkauskas et al., 2015; Jolivel et al., 2015; Dudvarski Stankovic et al., 2016). However, further studies using microglia/macrophage-specific GRP78-null or over-expressing mice are needed to determine whether GRP78 induction in activated microglia and macrophages indeed reflects its potential functions.

Our data revealed that vessel-associated GRP78 expression could be related to BBB impairment in the striatal lesion. Indeed, BBB breakdown in the striatum has been reported previously in rat models of 3-NP-induced Huntington’s disease (Sarkar and Schmued, 2010; Duran-Vilaregut et al., 2011). Our data also showed that some of GRP78/Iba1 double-labeled cells with amoeboid macrophage-like phenotypes were often localized in the vicinity of blood vessels, and that they expressed CD45, which is a marker of blood-derived macrophages (Guillemin and Brew, 2004). Given that microgliosis in the ischemic area is mainly due to proliferating resident microglia and not infiltrating macrophages (Li et al., 2013), GRP78/Iba1/CD45 triple-labeled cells, corresponding to only a small fraction of the GRP78/Iba1 double-labeled cells, might be infiltrating macrophages, and they may presumably infiltrate the lesion core through the compromised BBB induced by 3-NP. Moreover, the extravasation of the established BBB permeability marker FITC-albumin (Michalski et al., 2010; Krueger et al., 2015) was exclusively detectable in the lesion core, where astrocytes are virtually absent, but not in the peri-lesional area. In particular, vascular induction of GRP78 expression in the lesion core at days 3 and 7 days after 3-NP injection was coincident with the time and distribution of BBB leakage. Thus, vascular expression of GRP78 was correlated temporally and spatially with focal BBB breakdown and concomitantly occurring recruitment of activated microglia/macrophages induced by 3-NP. Considering that increased GRP78 could act as an inhibitor of macrophage adhesion (Bai et al., 2014) and enhance phagocytosis of amyloid-β peptides by microglia (Kakimura et al., 2001), GRP78 induction in activated microglia/macrophages might play a role in the activation/recruitment of these cells to the injured striatum, although the exact role played by GRP78 remains unclear.

A growing body of evidence suggests a novel role for GRP78 in the regulation of the BBB, despite its main function as a cellular chaperone protein. Alteration of GRP78 expression in vascular endothelial cells occurs in response to BBB disruption following subarachnoid hemorrhage (Yan et al., 2011). GRP78, associated with its cofactor HTJ-1, can be translocated and anchored to the cell surface by oxidized phospholipids, suggesting that GRP78 is a novel receptor initiating a protective vascular barrier response to oxidized phospholipids (Birukova et al., 2014). By contrast, lead exposure induces GRP78 expression in brain endothelial cells, which leads to Src activation and subsequent reduction of tight junctional proteins, with ensuing BBB disruption (Song et al., 2014). In addition, a recent study demonstrated that exposure to GRP78-specific recombinant antibodies results in increased BBB leakage, suggesting that GRP78 autoantibodies are associated with BBB disruption in neuromyelitis optica (Shimizu et al., 2017). Collectively, these results, including our present findings, suggest that GRP78 expression may be related to the development of BBB impairment in rats treated with 3-NP; however, the precise role of GRP78 in this process remains unclear. In addition, it remains to be clarified whether the increased GRP78 expression is a cause or a consequence of the BBB impairment. Thus, additional experiments, including two-photon microscopy for temporal and spatial *in vivo* imaging of BBB disruption, are required to clarify the role of GRP78 in BBB impairment.

Despite the potential relationship between GRP78 expression and BBB integrity, little is known about vessel-associated cells expressing GRP78 in the lesion core. Although a limited number of studies have reported GRP78 expression in vascular endothelial cells (Yan et al., 2011; Birukova et al., 2014; Song et al., 2014), to our knowledge, this is the first study to show that, in addition to endothelial cells, smooth muscle cells and adventitial cells also express GRP78. In particular, adventitial cells had the following features that were similar to those of perivascular fibroblast-like cells, reported in our previous study, although their exact cellular identity had not been elucidated: they were localized along the outer part of smooth muscle cells, had euchromatic nuclei with prominent nucleoli, and coexpressed GRP78 with nestin (Shin et al., 2013). A correlative approach using light and electron microscopy clearly demonstrated that, in these vasculature-associated cells, conspicuous silver-enhanced grains indicative of GRP78 were specifically localized in the rough ER/perinuclear cisternae, indicating that despite the phenotypic heterogeneity of vascular GRP78-positive cells, GRP78 was present as an ER protein in these cells. This ER localization of GRP78 in endothelial cells is unexpected considering that GRP78 is redistributed from the ER to the cytosol or plasma membrane of endothelial cells *in vitro*, where it acts as a receptor mediating for controlling cell viability and signaling (Birukova et al., 2014; Song et al., 2014). In our previous studies, however, GRP78 protein was almost exclusively localized to the rough ER in both constitutive cells (neurons) and cells induced by ER stress (reactive astrocytes and brain macrophages) after focal cerebral ischemia (Jin et al., 2018a). Interestingly, cells capable of GRP78 induction, such as activated microglia/macrophages, reactive astrocytes, and vessel-associated cells, particularly fibroblast-like adventitial cells, shared the following characteristics: they underwent marked morphological changes distinct from the cells in the control rats, and they had well-developed rough ER with prominent cisternal dilation, implying that protein synthesis was actively occurring in these cells. Thus, the previous findings and our observations indicate that although GRP78-positive cells in striatal lesions may represent a heterogeneous population of cells with functional diversity, GRP78 act as an ER-localized chaperone. However, further investigation is needed to determine the reason for GRP78 induction in these vascular cells, and to ascertain whether increased GRP78 expression reflects a specific function for this protein in the lesion core.

In summary, our data showed that constitutive GRP78 immunoreactivity was almost exclusively localized to striatal neurons, but it was induced in association with activated microglia/macrophages and reactive astrocytes in the striata of rats treated with 3-NP. In addition, GRP78 was induced in cells of the vascular wall, including endothelial cells, smooth muscle cells, and adventitial fibroblast-like cells, in which GRP78 protein was specifically localized in the rough ER/perinuclear cisternae. The vascular induction of GRP78 appeared to correlate temporally and spatially with infiltration of activated microglia into the lesion core, and coincided with BBB leakage, indicating that the expression of GRP78 was induced in vessels with BBB impairment in the lesion core. Thus, our data further support the link between GRP78 expression and an activated functional status of neuroglial cells during CNS insults, and provide a novel insight into the phenotypic and functional heterogeneity of GRP78-positive cells, suggesting GRP78 involvement in the activation/recruitment of activated microglia/macrophages and its potential role in the BBB impairment that occurs in response to neurotoxic insults such as 3-NP.

## Author Contributions

All authors have contributed significantly to the research and the article preparation. XJ contributed to the treatment of the experimental animals, immunohistochemistry, immunoelectron microscopy, and quantitative analysis. T-RR and SK worked on the treatment of the experimental animals and immunohistochemistry. HK worked on the electron microscopy. M-YL worked on the design of the study, data analysis, and final manuscript preparation.

## Conflict of Interest Statement

The authors declare that the research was conducted in the absence of any commercial or financial relationships that could be construed as a potential conflict of interest.
